# The Complete Mitogenome of the Estuarine Clam *Potamocorbula amurensis* (Corbulidae, Myida) and Its Implications for Phylogeny and Adaptation

**DOI:** 10.1002/ece3.73937

**Published:** 2026-06-30

**Authors:** Xuyi Yang, Yalin Feng, Liang Xu, Zan Liu, Tinghui Xie, Zefeng Yu, Junyuan Li

**Affiliations:** ^1^ College of Agriculture and Bioengineering Taizhou Vocational College of Science and Technology Taizhou China; ^2^ Chimin Health Management co., Ltd. Taizhou China

**Keywords:** adaptive evolution, Mitogenome, molecular phylogeny, positive selection, *Potamocorbula amurensis*

## Abstract

*Potamocorbula amurensis* is a euryhaline bivalve of considerable ecological and economic importance in estuarine ecosystems of the North Pacific. However, the absence of a well‐characterized mitogenome has limited a comprehensive understanding of phylogenetic relationships within order Myida, as well as the genomic mechanisms underlying its adaptation to estuarine environments. In this study, the complete mitogenome of *P. amurensis* was sequenced and assembled, which consists of a 21,044 bp circular molecule encoding 13 protein‐coding genes, 2 rRNA genes, and 23 tRNA genes, and exhibits a pronounced A + T bias (68.18%). Notably, the otherwise frequently undetected ATP synthase F0 subunit 8 (*atp8*) gene was successfully identified and annotated, confirming its presence in the family Corbulidae. Phylogenetic analyses revealed that Corbulidae, represented by *P. amurensis* , forms a sister clade to the Myidae–Dreissenidae lineage with strong nodal support. The estimation of divergence times indicated that *P. amurensis* diverged during the Middle Jurassic, coinciding with major marine diversification events. Furthermore, signatures of positive selection were detected in nad5 along the *P. amurensis* lineage, suggesting mitochondrial adaptation in oxidative phosphorylation pathways in response to osmotic and thermal variability in estuarine environments. Collectively, these findings provide key insights into the mitogenomic evolution and estuarine adaptation of *P. amurensis* , and may inform future conservation strategies for this ecologically and economically important species.

## Graphical abstract

This study reports the first complete mitogenome of the estuarine clam *Potamocorbula amurensis*, revealing its phylogenetic position as a sister group to the Myidae–Dreissenidae clade. Molecular dating indicates a Middle Jurassic divergence, and positive selection was detected in the nad5 gene, suggesting mitochondrial adaptations to osmotic stress in estuarine habitats.

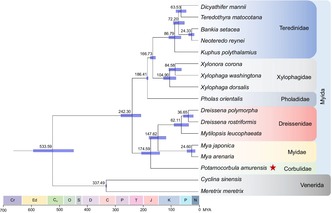

## 1 Introduction

The class Bivalvia represents a highly diverse and ecologically significant group of mollusks that play essential roles in aquatic ecosystems, functioning as filter feeders, agents of bioturburation, and integral components of benthic trophic networks (Taylor et al. 2007; Plazzi et al. 2011). Within this class, the order Myida which is comprised of several families that are evolutionarily adapted for an infaunal, sediment‐burrowing mode of life, with representative taxa exhibiting remarkable physiological plasticity in response to salinity gradients (Combosch et al. 2017). The family Corbulidae exemplifies this pronounced environmental adaptability, characterized by small, markedly inequivalve shells, and an exceptional tolerance to brackish‐water conditions (Hallan et al. 2013). The euryhaline bivalve *Potamocorbula amurensis* (Schrenck, 1861) is a prominent member of the family Corbulidae, with a broad distribution across marine and estuarine habitats of the northern Pacific Ocean, ranging from the Russian Far East to Japan, Korea, and China (Coan 2002; Huber 2013). As a keystone species in aquatic ecosystems, *P. amurensis* plays a critical ecological role as a key component of benthic trophic networks and as an important live feed resource in shrimp and crab aquaculture (Poulton et al. 2002). In certain coastal regions, this species is processed into traditional seafood products, highlighting its considerable economic significance (Sun et al. 2014). Ecologically, *P. amurensis* is well adapted to dynamic intertidal and subtidal environments, where it inhabits and burrows within muddy substrates and tolerates wide salinity fluctuations ranging from near‐freshwater to fully marine conditions. This broad physiological tolerance enables the species to occupy transitional habitats that are typically unsuitable for stenohaline taxa (Carlton et al. 1990). However, in recent decades, *P. amurensis* populations have experienced intensifying pressures from environmental pollution, habitat degradation, and climate change (Stewart et al. 2013). Notably, the biomass of *P. amurensis* in the estuarine regions of China has declined by more than 60% over the past two decades (Cai et al. 2016). Paradoxically, despite this regional decline within its native range in Asia, *P. amurensis* has emerged as a highly successful invasive species. In particular, following its introduction via ballast water in the 1980s, the species rapidly established and subsequently became a dominant component of benthic communities in San Francisco Bay, California (Pereira et al. 1992). This apparent paradox highlights the urgent need to elucidate the biological and genetic determinants underlying the persistence and competitive success of *P. amurensis* , a knowledge essential for guiding effective conservation in its native habitat and control measures in its invasive range (Miller et al. 2014).

Mitochondrial genomes (mitogenomes) have become integral to molecular evolutionary research, providing a rich source of information for phylogenetic inference, population genetics analyses, and the elucidation of molecular adaptation (Yang et al. 2021; Zhao et al. 2022; Ma et al. 2023). The mitogenomes of several bivalve lineages exhibit doubly uniparental inheritance (DUI), a distinctive system in which males harbor two distinct mitogenomes (M‐type and F‐type) that can undergo significant divergence (Theologidis et al. 2008; Ghiselli et al. 2021). This phenomenon, documented in orders such as Myida, provides a valuable framework for investigating sex determination mechanisms and mitochondrial recombination, with recent studies identifying recombination events in DUI‐associated mitogenomes that may shape evolutionary trajectories (Smith et al. 2023). Mitogenomic analyses within the order Myida, which comprises diverse families adapted to a wide range of marine and estuarine habitats, have revealed gene rearrangements, nucleotide composition biases, and signatures of selection potentially associated with ecological specialization (Plazzi et al. 2016; Ghiselli et al. 2021). Despite these advances, the mitogenome of *P. amurensis* , a representative member of the family Corbulidae and an estuarine specialist, remains uncharacterized to date. This knowledge gap hinders a comprehensive resolution of the phylogenetic relationships within Myida, particularly regarding the contentious systematic placement of Corbulidae relative to families such as Myidae and Dreissenidae (Taylor et al. 2007; Bieler et al. 2014; Lemer et al. 2019). Furthermore, estuarine environments impose distinct selective pressures, including salinity fluctuations and osmotic stress, which may drive adaptive evolution in mitochondrial genes associated with energy metabolism (Tomasco and Lessa 2011; Zhao et al. 2022). Investigating these processes in *P. amurensis* may therefore yield key insights into the mechanisms underlying the adaptation of bivalves to highly dynamic habitats in the context of ongoing global environmental change.

In this study, the complete mitogenome of *P. amurensis* collected from the Jiaojiang Estuary was sequenced and characterized. Its phylogenetic position within Myida was reconstructed, the lineage divergence times were estimated, and comprehensive selection analyses were conducted to detect signatures of adaptive molecular evolution potentially associated with its estuarine lifestyle. Collectively, the results provide novel insights into the genomic basis of adaptation to brackish environments in Corbulidae and highlight the broader evolutionary significance of mitochondrial variation in mediating responses to osmotic stress.

## 2 Materials and Methods

### 2.1 Specimen Collection and DNA Extraction

Specimens of *P*. *amurensis* (Figure S1; *n* = 16) were collected from the Jiaojiang Estuary, Taizhou, China (121°24′50.38″ E, 28°41′4.9″ N) on 20 July 2024 and either frozen at −80°C for DNA extraction or preserved in 95% ethanol for morphological identification. Genomic DNA was extracted from adductor muscle tissues using the sodium dodecyl sulphate (SDS) method. The quantity and quality of the extracted DNA were assessed by agarose gel electrophoresis and a Qubit Fluorometer (Invitrogen, USA), respectively.

### 2.2 Mitogenome Sequencing, Assembly, and Annotation

A whole‐genome Illumina sequencing strategy was employed to obtain the complete mitogenome sequence of *P. amurensis* . Briefly, 1 μg of high‐molecular‐weight genomic DNA was used to construct paired‐end libraries. Genomic DNA was purified using the TIANgel Midi Purification Kit (Tiangen Biotech Co. Ltd., Beijing, China) following the manufacturer's instructions and subsequently fragmented to ~350 bp insert sizes using a Covaris M220 system. Sequencing libraries were constructed using the NEBNext Ultra DNA Library Prep Kit for Illumina (New England Biolabs, Ipswich, MA, England) according to the manufacturer's instructions. The quality‐checked paired‐end libraries were sequenced on an Illumina NovaSeq 6000 platform (Biozeron, Shanghai, China). The raw reads were processed using fastp to remove low‐quality reads and adaptor‐contaminated sequences (Chen et al. 2018) using the following parameters: ‐q 10 ‐u 50 ‐y ‐g ‐Y 10 ‐e 20 ‐l 100 ‐b 150 ‐B 150. In total, 6.64 Gb of high‐quality clean reads were retained for downstream mitogenome assembly.

The clean reads were used for *de novo* assembly using GetOrganelle v1.7.7.0 (Jin et al. 2020) with the following parameters: ‐R 10 ‐k 21,45,65,85,105 ‐F animal_mt. Putative contigs were identified by alignment against protein‐coding genes (PCGs) in the mitogenome database (http://ftp.ncbi.nlm.nih.gov/refseq/release/mitochondrion/) using the locally installed NCBI BLAST tool (version 2.8.1+; Camacho et al. 2009). Finally, The complete mitogenome sequence of *P. amurensis* was obtained, and the PCGs, transfer RNA genes (tRNAs), and ribosomal RNA genes (rRNAs) were annotated using the MITOS2 web server (Bernt et al. 2013) with the invertebrate mitochondrial genetic code. Gene boundaries were further refined by comparison with closely related mitogenome sequences retrieved from NCBI using MEGAX (Kumar et al. 2018). Base composition and relative synonymous codon usage (RSCU) were calculated using PhyloSuite v1.2.3 (Zhang et al. 2020). The AT‐ and GC‐skews were computed using the following formulae: AT‐skew = (A − T)/(A + T) and GC‐skew = (G − C)/(G + C) (Perna and Kocher 1995). The mitogenome of *P. amurensis* was visualized using OGDRAW v1.3.1 with default parameters (Greiner et al. 2019).

### 2.3 Phylogenetic Analysis and Estimation of Divergence Time

To elucidate the phylogenetic position of *P. amurensis* within the order Myida, phylogenetic analyses were conducted using a concatenated mitochondrial dataset comprising 12 shared PCGs from 15 species within Myida, with two Venerida species (*Cyclina sinensis* and *Meretrix meretrix*) included as outgroups. The nucleotide sequences of the 12 PCGs were extracted, aligned using MAFFT v7.310 (Katoh and Standley 2013), and trimmed with TrimAl v1.4 (Capella‐Gutierrez et al. 2009) prior to concatenation. The suitability of the nucleotide dataset for phylogenetic reconstruction was evaluated through substitution saturation analyses using DAMBE7 (Xia et al. 2003). Assuming a symmetrical tree topology, the observed index of substitution saturation (Iss = 0.6352) was significantly lower than the corresponding critical threshold (Iss.c = 0.8417, *p* < 0.0001; Table S1), indicating little to no substitution saturation and thereby confirming that the dataset retains a reliable phylogenetic signal. The optimal partitioning schemes and corresponding nucleotide substitution models were determined using ModelFinder v2.2.0 (Kalyaanamoorthy et al. 2017), based on the corrected Akaike information criteria (AICc). The best‐fitting substitution models identified for each partition are provided in Table S2. A maximum likelihood (ML) phylogenetic tree was constructed using IQ‐TREE v2.2.0 (Nguyen et al. 2015) under the selected partitioning scheme with 1000 ultrafast bootstrap replicates. Bayesian Inference (BI) phylogenetic analysis was performed using MrBayes v3.2.1 (Ronquist et al. 2012), applying the same partitioning strategy and substitution models. Two independent Markov chain Monte Carlo (MCMC) runs were performed, each comprising four chain and 2 × 10^6^ generations, with sampling every 100 generations. To further verify convergence, an additional analysis was conducted using two independent runs of 5 × 10^6^ generations. The initial 25% of samples were discarded as burn‐in, following standard practice in MCMC‐based phylogenetic inference. Convergence was assessed by comparing posterior estimates, which showed < 1% variation in mean node ages and strongly overlapping 95% highest posterior density (HPD) intervals, indicating satisfactory convergence. The resulting ML and BI trees were visualized using iTOL v5 (https://itol.embl.de) (Letunic and Bork 2021).

To estimate the divergence times of *P. amurensis* , 12 shared PCGs from 15 Myida species and two Venerida species were concatenated and aligned. The multi‐gene alignment was partitioned into 1st, 2nd and 3rd codon positions using PhyloSuite v1.2.3 (Zhang et al. 2020). ML phylogenetic trees were reconstructed in PhyML v3.1 (Guindon et al. 2010) under the best‐fitting model (TVM + F + I + G4), with branch support evaluated using 100 bootstrap replicates. The divergence times were estimated using the approximate likelihood method implemented in MCMCTree (PAML v4.8j; Yang 2007), incorporating two fossil calibration constraints (Table S3). Prior parameters for the rgene_gamma distribution were derived from the overall substitution rate estimated from the ML phylogenetic tree inferred using BASEML in the PAML package. The gradient vector and Hessian matrix of branch lengths were computed in BASEML under the GTR + G substitution model (dos Reis and Yang 2011). MCMCTree analyses were performed under an independent‐rates molecular clock model (clock = 2) and the GTR + G substitution model. The model settings included a substitution rate per time unit of 0.080406, with rgene_gamma = (1, 12.5) and sigma2_gamma = (1, 4.5). Convergence was assessed using two independent MCMC runs of 5 × 10^6^ generations, with the initial 10^4^ generations discarded as burn‐in.

### 2.4 Analyses of Selection Pressure

The ratio of nonsynonymous (dN) to synonymous (dS) substitution rates (ω = dN/dS) is widely used as an indicator of selection pressure acting on PCGs, where ω = 1 indicates neutral evolution, ω < 1 indicates purifying selection, and ω > 1 indicates positive selection (Biswas and Akey 2006). Variation in ω among amino acid sites reflects heterogeneous selection pressures and is widely regarded as a key indicator of adaptive evolution in species (Fay and Wu 2003). To investigate the potential signatures of adaptive evolution in the mitochondrial genes of *P. amurensis* from the estuarine regions, we performed positive selection analyses using the CodeML module in PAML v4.9j (Yang 2007). The phylogenetic tree inferred from the 12 PCGs using PhyML was used as the input topology for the selection analyses. A free‐ratios model (branch model) was initially implemented to estimate lineage‐specific average dN/dS ratios (ω) across the 12 PCGs, which were used to characterize the evolutionary rates of the mitogenomes. The one‐ratio model (model = 0, NSsites = 0, icode = 4) and the two‐ratio model (model = 2, NSsites = 0, icode = 4) were subsequently applied to evaluate the differences in ω values among predefined branches in the phylogenetic tree. The *P. amurensis* clade inhabiting estuarine regions was designated as the foreground branch, and the remaining clades were treated as background branches. In addition, branch‐site models (model = 2, NSsites = 2) were independently implemented for each PCG to detect signatures of positive selection at specific codon sites along the foreground lineage. Positively selected sites were identified using the Bayes Empirical Bayes (BEB) approach, with posterior probabilities > 0.95. Likelihood ratio tests (LRTs) were additionally performed to assess whether the alternative models provided a significantly better fit to the data than their corresponding null models.

To further elucidate the functional significance of the putatively positively selected sites, three‐dimensional protein structures were predicted using AlphaFold 3 (Abramson et al. 2024), and the highest‐scoring model was selected for structural analysis and visualization in PyMOL v3.1.6.1 (DeLano 2002).

## 3 Results and Discussion

### 3.1 Features of the *P. amurensis* Mitogenome

**FIGURE 1:**
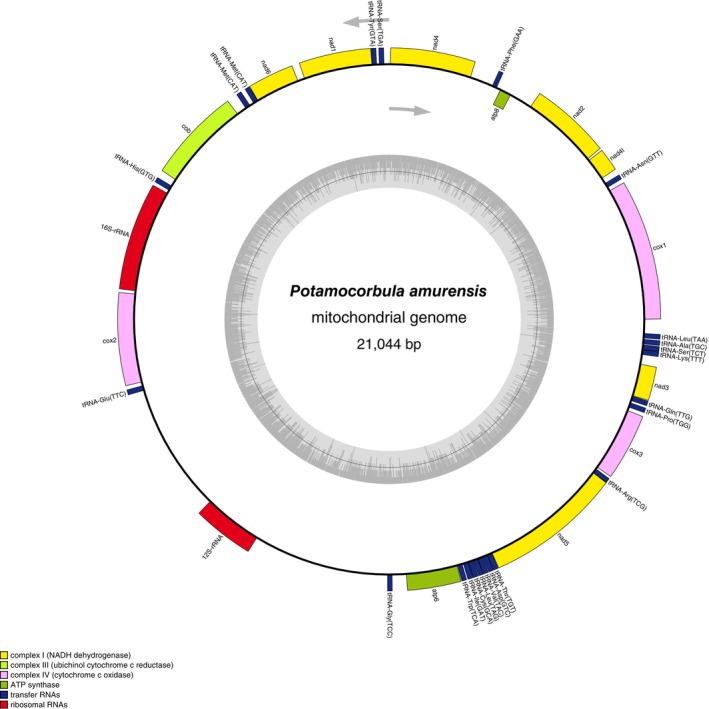
The mitogenome of *P. amurensis* . Annotated genes are colored according to the functional categories. Genes on the outside are transcribed in the clockwise direction, whereas genes on the inside are transcribed in the counterclockwise direction.

The complete mitogenome of *P. amurensis* is a circular DNA molecule of 21,044 bp (GenBank accession ID: PX644768) (Figure 1). This genome size is smaller than that of *Mya japonica* , but exceeds those reported for other species within Myida (Table 1). The intergenic regions span 5546 bp, accounting for 26.35% of the mitogenome, which represents a significantly higher proportion than that observed in other taxa within Myida. This elevated proportion of non‐coding DNA may encompass multiple putative control regions or duplicated genomic segments that could contribute to the initiation of replication and transcriptional regulation. Similar genomic architectures associated with mitogenomic expansion have been reported in other bivalve taxa, such as *M. japonica* , which harbors five D‐loop regions (Dai et al. 2024).

**TABLE 1:** General features of mitogenomes in the order Myida.

Genome features	*Potamocorbula amurensis*	*Mya arenaria*	*Mya japonica*	*Dreissena polymorpha*	*Pholas orientalis*	*Xylonora corona*	*Teredothyra matocotana*	*Bankia setacea*
Genome size (bp)	21,044	18,177	21,396	18,452	18,995	15,083	17,364	16,986
GC content (%)	31.82	34.65	33.60	35.12	33.10	42.90	37.91	39.80
rRNA/tRNA/PCGs/Total	2/23/13/38	2/23/12/37	2/23/13/38	2/22/12/36	2/23/12/37	2/23/12/37	2/23/12/37	2/23/12/37
PCG total length (bp)	11,841	12,030	12,768	12,636	11,907	10,717	11,865	11,620
PCG average length (bp)	911	1002	982	1053	992	893	989	968
PCG's GC content (%)	32.15	37.59	30.77	35.23	30.48	44.49	38.71	39.68
% of genome (PCGs)	56.27	66.18	59.67	68.48	62.68	71.05	68.33	68.41
Intergenic region length (bp)	5546	2227	4734	2317	3376	949	1894	1682
AT‐skew/GC‐skew	−0.262/0.360	−0.129/0.315	−0.122/0.352	0.069/−0.127	−0.240/0.335	−0.249/0.233	−0.294/0.370	−0.233/0.305
GenBank accession	PX644768	MW727516	OR502892	KY091877	OQ858578	OM910828	NC_063767	OM910805

The mitogenome of *P. amurensis* consists of 13 PCGs, 2 rRNAs, and 23 tRNAs. Out of 38 annotated genes, 37 are encoded on the heavy strand, whereas only the ATP synthase F_0_ subunit 8 (*atp8*) gene is located on the light strand (Figure 1; Table 1). The exclusive localization of the *atp8* gene on the light strand, in contrast to the heavy‐strand orientation of the remaining genes, is particularly noteworthy, as bivalve mitogenomes generally exhibit a strong strand bias in gene encoding, with all PCGs typically transcribed from a single strand. This strand‐specific localization of *atp8* is consistent with patterns commonly observed in the M‐type mitogenomes of bivalves exhibiting DUI (Breton et al. 2007; Passamonti et al. 2011; Ghiselli et al. 2021). In species exhibiting DUI, the M genome frequently undergoes gene‐order rearrangements and occasional gene inversions, potentially altering the transcriptional orientation of individual genes relative to that of the maternally transmitted F genome. The gene content and overall organization of the sequenced mitogenome of *P. amurensis* are consistent with those of a singly uniparental F‐type‐like mitogenome, but the genome harbors an *atp8* gene with an M‐type‐like orientation. This observation raises the possibility that the sequenced mitogenome represents an M‐type genome or a recombinant molecule resulting from occasional recombination events reported between F‐ and M‐type mitogenomes in bivalves exhibiting DUI (Smith et al. 2023). Future studies using sex‐specific tissues or pedigree analyses are necessary for determining whether *P. amurensis* exhibits DUI and whether the light‐strand localization of *atp8* is characteristic of the M genome.

Furthermore, the absence of *atp8* has been documented in multiple bivalve lineages (Wilson et al. 2015; Uliano‐Silva et al. 2016; Soroka et al. 2018). In this study, the *atp8* gene was manually annotated in the assembled mitogenome and identified as a 156 bp open reading frame. This finding represents only the second reported annotation of *atp8* in Myida species, following its initial identification in the soft‐shell clam *M. japonica* (Dai et al. 2024). Emerging evidence suggests that the apparent absence of *atp8* may reflect limitations in genome annotation rather than true gene loss, as this gene is characterized by high sequence divergence and considerable variation in length across taxa. Consequently, *atp8* is frequently overlooked by automated annotation pipelines, and its reliable identification often requires manual curation supported by comparative sequence alignment with orthologous genes from closely related species (Zhao et al. 2022; Dai et al. 2024). Our results provide evidence for the presence of the *atp8* gene in Corbulidae. While this finding does not indicate its conservation across the entire Myida order, it provides unequivocal evidence for its occurrence in at least one family within the group. However, further investigations are warranted to confirm the transcriptional activity of this gene and to elucidate its functional significance (Lubośny et al. 2018).

**FIGURE 2:**
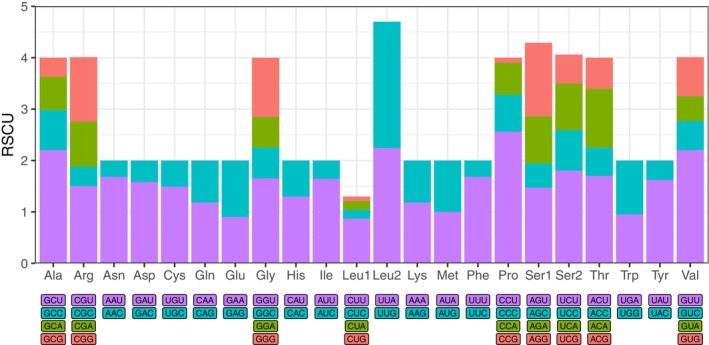
RSCU of the *P. amurensis* mitogenome. Numbers to the left refer to the total number of the RSCU values. Codon families are plotted on the x‐axis.

The mitogenome exhibited a nucleotide composition of 25.16% A, 43.02% T, 10.18% C, and 21.64% G, yielding an A + T content of 68.18%, which significantly exceeded the C + G content (31.82%; Table 1). The *P. amurensis* mitogenome exhibited a pronounced compositional bias characterized by a negative AT skew (−0.262) and a positive GC skew (0.360). These patterns of base composition and nucleotide asymmetry are consistent with those commonly reported in studies on bivalve mitogenomes (Wilson et al. 2015; Soroka et al. 2018; Yang et al. 2019; Dai et al. 2024). RSCU analysis of the PCGs in the *P. amurensis* mitogenome revealed that 24 codons exhibited RSCU values greater than 1.00, of which 19 terminated in either A or U, suggesting a pronounced A/T bias in the third codon position (Figure 2; Table S4). This finding supports the hypothesis that A/T bias at the third codon position in mitogenomes may positively correlate with codon usage bias (Kou et al. 2020; Ma et al. 2023). All the PCGs in the *P. amurensis* mitogenome initiated with the typical ATN codon, except for *nad4* and *cox2*, which utilized TTG as the initiation codon. Five PCGs, namely, *nad4*, *nad5*, *nad6*, *cox2*, and *cox3*, terminated with the TAG stop codon, whereas the remaining genes terminated with the TAA codon (Table 2). Additionally, a single nucleotide overlap (AG) was identified between *nad5* and *trnR* in the *P. amurensis* mitogenome.

**TABLE 2:** The organization of the mitogenome of *P. amurensis* .

Gene	Location		Codon		Length (bp)	Strand	Intergenic nucleotides^a^
	From	To	Start	Stop			
*cox1*	1	1770	ATA	TAA	1770	+	48
*trnN(gtt)*	1819	1883			65	+	76
*nad4l*	1960	2253	GTG	TAA	294	+	23
*nad2*	2277	3296	ATA	TAA	1020	+	300
*atp8*	3597	3752	ATG	TAA	156	−	54
*trnF(gaa)*	3807	3869			63	+	306
*nad4*	4176	5246	TTG	TAG	1071	+	80
*trnS(tga)*	5327	5392			66	+	30
*trnY(gta)*	5423	5485			63	+	0
*nad1*	5486	6394	ATG	TAA	909	+	89
*nad6*	6484	7071	ATG	TAG	588	+	0
*trnM(cat)*	7072	7137			66	+	54
*trnM(cat)*	7192	7260			69	+	129
*cob*	7390	8583	ATT	TAA	1194	+	95
*trnH(gtg)*	8679	8746			68	+	42
*rrnL*	8789	10,148			1360	+	38
*cox2*	10,187	11,353	TTG	TAG	1167	+	46
*trnE(ttc)*	11,400	11,468			69	+	1711
*rrnS*	13,180	13,958			779	+	1801
*trnG(tcc)*	15,760	15,824			65	+	182
*atp6*	16,007	16,684	ATT	TAA	678	+	4
*trnW(tca)*	16,689	16,755			67	+	11
*trnI(gat)*	16,767	16,838			72	+	0
*trnC(gca)*	16,839	16,904			66	+	2
*trnL(tag)*	16,907	16,970			64	+	0
*trnV(tac)*	16,971	17,036			66	+	2
*trnD(gtc)*	17,039	17,104			66	+	0
*trnT(tgt)*	17,105	17,169			65	+	1
*nad5*	17,171	18,886	ATG	TAG	1716	+	−2
*trnR(tcg)*	18,885	18,949			65	+	24
*cox3*	18,974	19,822	ATT	TAG	849	+	44
*trnP(tgg)*	19,867	19,931			65	+	25
*trnQ(ttg)*	19,957	20,022			66	+	3
*nad3*	20,026	20,454			429	+	127
*trnK(ttt)*	20,580	20,645			66	+	0
*trnS(tct)*	20,646	20,711			66	+	7
*trnA(tgc)*	20,719	20,784			66	+	12
*trnL(taa)*	20,797	20,861			65	+	182

^a^ The positive number indicates interval base pairs between genes, while the negative number indicates the overlapping base pairs between genes.

### 3.2 Phylogenetic Analysis and Divergence Time Estimation of *P. amurensis*

A concatenated 10,557 bp nucleotide alignment was generated from 12 shared PCGs across the mitogenomes of 15 species within Myida; additionally, *Cyclina sinensis* (Venerida) and *Meretrix meretrix* (Venerida) were selected as outgroups. This concatenated alignment was used to infer the phylogenetic position of *P. amurensis* within the order Myida using ML and BI methods. Both methods yielded congruent topologies with consistently high support values, in agreement with previously published phylogenetic studies (Lemer et al. 2019; Wang et al. 2023). Phylogenetic tree analyses revealed that 15 Myida species clustered into six well‐supported clades corresponding to the families Teredinidae, Xylophagidae, Pholadidae, Dreissenidae, Myidae, and Corbulidae. The family Corbulidae represented by *P. amurensis* forms a sister group to the common ancestor of Myidae and Dreissenidae, with strong bootstrap support values (Figure S2). The phylogenetic position of the Corbulidae family remains contentious. Traditional morphological analyses, as well as certain molecular phylogenetic studies based on 18S and 28S rRNA genes, have recovered Corbulidae and Myidae as sister groups (Taylor et al. 2007; Bieler et al. 2014). However, the present study clearly supports Corbulidae as a sister lineage to the clade comprising Myidae and Dreissenidae. Furthermore, the extensive fossil record of Corbulidae extends back to the Lower Jurassic, whereas Myidae appears later in the Lower Cenozoic (Skelton and Benton 1993). Collectively, these data support a more complex evolutionary history within Myida, in which the lineage comprising Myidae and Dreissenidae diverged from Corbulidae.

**FIGURE 3:**
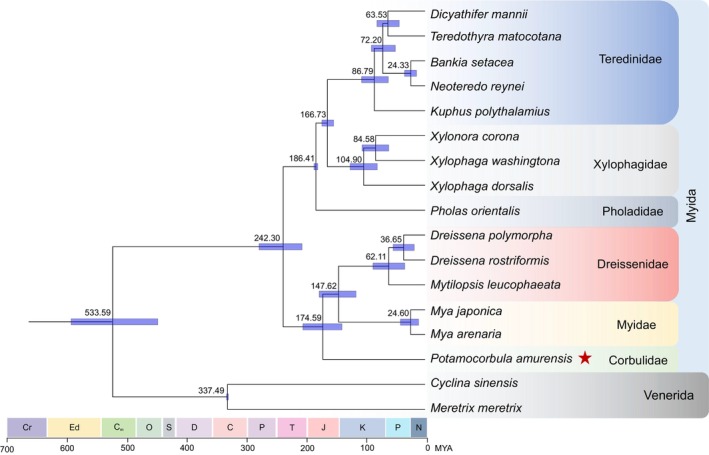
Posterior estimates of divergence time of 17 taxa on the phylogenetic tree. Blue bars depict the 95% highest posterior density (HPD) and the values at the nodes represent posterior mean ages. Estimations were performed with MCMCTree based on the independent rate model using two fossil calibrations on nodes. C, Carboniferous; Cm, Cambrian; Cr, Cryogenian; D, Devonian; Ed, Ediacaran; J, Jurassic; K, Cretaceous; N, Neogene; O, Ordovician; P, Palaeogene; P, Permian; S, Silurian; T, Triassic.

A time‐calibrated phylogeny was reconstructed for 17 bivalve species based on a concatenated alignment of 12 PCGs (Figure 3). The basal diversification of Venerida was calibrated to 339.4–336 Ma (Mega‐annum) based on the fossil record of *Pullastra striatocostata* (M'Coy 1847; Wang et al. 2023), whereas the crown node of Pholadidae was constrained to 189.6–183 Ma using fossil evidence from *Martesia* sp. (Velazco 2008). Independent ML runs yielded highly similar topologies, indicating that the analyses had reached convergence (dos Reis and Yang 2011). Molecular clock analyses suggested that *P. amurensis* diverged from the common ancestor of Myidae and Dreissenidae approximately 174.59 Ma (95% HPD: 141.85–208.48 Ma) during the Middle Jurassic (Figure 3). Furthermore, the estimated divergence times for the families Pholadidae (~186.41 Ma), the Xylophagidae–Teredinidae clade (~166.73 Ma), and the Myidae–Dreissenidae clade (~147.62 Ma) all fall within the Middle to Late Jurassic. This temporal clustering of speciation events coincides with a period of major marine diversification characterized by rising sea levels and the formation of extensive warm, shallow epicontinental seas (Korte et al. 2015). These newly available habitats, coupled with intensified predation pressure, likely promoted ecological adaptation, diversification, and niche partitioning among marine invertebrates (Hao et al. 2025). The estuarine niche of *P. amurensis* , characterized by dynamic, brackish‐water conditions, imposes distinct physiological challenges. These conditions likely acted as a key driver of its genetic isolation and subsequent divergence from the fully marine lineages of Myidae and Dreissenidae. Therefore, the speciation of *P. amurensis* is not an isolated event, but rather reflects broader macroevolutionary processes that shaped bivalve diversity during this era.

### 3.3 Adaptive Molecular Evolution

Estuarine environments are characterized by highly dynamic abiotic conditions, including salinity fluctuations, temperature variability, and osmotic stress, in contrast to the relatively stable conditions of marine habitats (Zhang et al. 2015). These stressors exert strong selective pressures on estuarine organisms such as *P. amurensis* , potentially driving adaptive evolution in key genetic systems, including the mitogenome, which is central to cellular respiration and energy metabolism (Tomasco and Lessa 2011). To assess whether the estuarine lineage of *P. amurensis* has experienced positive selection relative to its marine relatives, we estimated the dN/dS ratios across 12 PCGs. Both branch models and branch‐site models implemented in PAML were applied, with the *P. amurensis* lineage designated as the foreground branch to detect signatures of adaptive evolution.

Under the branch models, the one‐ratio model (M_0_) implemented in PAML estimated a mean ω ratio of 0.0259 for the 12 concatenated mitochondrial PCGs across all 17 bivalve species, indicating strong purifying selection to maintain their functional integrity. The free‐ratio model (M_1_) provided a significantly better fit than the M_0_ (2ΔlnL = 1007.96, *p* < 0.001; Table S5), confirming substantial heterogeneity in selective pressures among bivalve lineages. The two‐ratio model (M_2_) also significantly outperformed M_0_ (2ΔlnL = 40.64, *p* < 0.001), with an elevated ω on the estuarine *P. amurensis* lineage (ω_1_ = 0.1640) relative to background branches (ω_0_ = 0.0258). A direct likelihood ratio test showed that the M_1_ fits the data significantly better than the M_2_ (2ΔlnL = 967.32, *p* < 0.001), indicating that selective pressures vary considerably among non‐foreground branches as well. Despite this heterogeneity, both models consistently recovered a markedly elevated ω value along the *P. amurensis* lineage relative to those of the background lineages. Taken together, these results provide robust evidence for a relaxation of purifying selection in this estuarine specialist.

**FIGURE 4:**
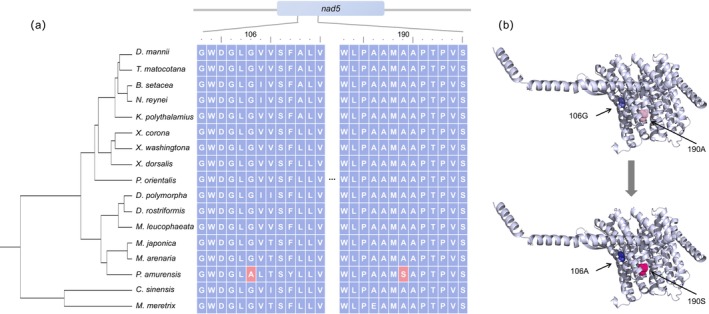
The structure analysis of *nad5* in *P. amurensis* . (a) Positively selected sites in the conserved regions of the *nad5* gene. (b) Structures adjacent to the substituted sites of the *nad5* gene in *P. amurensis* .

Positive selection typically acts on a limited number of sites over short evolutionary timescales and is often obscured by pervasive purifying selection acting across gene sequences (Zhang et al. 2005). BEB analysis identified the positively selected sites in *atp6*, *cytb*, *nad1*, *nad2*, *nad3*, *nad4*, *nad4l* and *nad5* in the *P. amurensis* lineage, all with posterior probabilities > 95%. However, only *nad5* showed statistically significant evidence of positive selection at the gene level, suggesting that it may play a key role in the adaptive evolution of *P. amurensis* in response to estuarine environments (Table 3). Specifically, two positively selected amino acid sites (G106A and A190S) were identified within the highly conserved region of the *nad5* gene of *P. amurensis* (Figure 4), and these substitutions may affect the structural flexibility and polarity of the encoded protein, potentially enhancing enzymatic function under challenging estuarine conditions. Successful adaptation to brackish estuarine environments may have required increased energy demand for processes such as osmoregulation (Caballero et al. 2015). NADH dehydrogenase, the largest and the most structurally complex enzyme of the mitochondrial respiratory chain, accepts electrons generated from NADH oxidation and transfers them to ubiquinone, thereby catalyzing its reduction to ubiquinol. The protein encoded by *nad5* is a core subunit of NADH dehydrogenase, which plays a pivotal role in proton translocation and the generation of the proton‐motive force, thereby coupling electron transfer to ATP synthesis (Bai et al. 2000). Mutation in subunits of NADH dehydrogenase may impair metabolic efficiency by altering electron transport activity, thereby potentially affecting organismal fitness (Zhang et al. 2021; Zhao et al. 2022). Therefore, the observed positive selection on specific sites within the *nad5* gene in *P. amurensis* could enhance the efficiency of the electron transport chain, enabling better energy production under osmotic stress conditions prevalent in estuaries. Positive selection on NADH dehydrogenase complex (Complex I) subunits has been reported in organisms facing osmotic challenges in low‐salinity or freshwater environments, For example, *nad2* in freshwater dolphins (Caballero et al. 2015), *nad4* in the freshwater mussel *Limnoperna fortunei* (Zhao et al. 2022), and *nad5* in the freshwater razor clam *Novaculina chinensis* (Meng et al. 2025). These recurrent changes, particularly in *nad*5, are regarded as key molecular adaptations that enhance mitochondrial efficiency and ATP production under the osmotic and energetic stresses imposed by hypo‐osmotic freshwater habitats, consistent with the positive selection on *nad5* we observed in the estuarine *P. amurensis* . The recurrent positive selection on the same NADH dehydrogenase subunits in phylogenetically distant lineages (bivalves and mammals) underscores the critical role of NAD genes in freshwater adaptation. The significant positive selection detected on *nad5* in the estuarine *P. amurensis* therefore aligns with this conserved pattern, suggesting that adaptive modifications in this core subunit represent a pivotal mechanism enabling organisms to cope with the elevated energy demands under fluctuating or low‐salinity environments.

**TABLE 3:** Branch‐site model analyses in *P. amurensis* branch.

Genes	Model	lnL	2∆lnL	Parameter estimates	Positive sites	*p*
*atp6*	Alternative	−6622.31	3.34	*P*_0_ = 0.214 *P*_1_ = 0.008 *P*_2a_ = 0.751 *P*_2b_ = 0.027 ω_0_ = 0.038 ω_1_ = 1.000 ω_2_ = 423.61	54G(0.966) 56 T(0.992) 119E (1.000) 130F(0.961) 137Y(0.990)	0.067
	Null	−6623.98		*P*_0_ = 0.266 *P*_1_ = 0.010 *P*_2a_ = 0.699 *P*_2b_ = 0.025 ω_0_ = 0.036 ω_1_ = 1.000 ω_2_ = 1.000		
*cytb*	Alternative	−7771.13	0	*P*_0_ = 0.266 *P*_1_ = 0.028 *P*_2a_ = 0.639 *P*_2b_ = 0.067 ω_0_ = 0.040 ω_1_ = 1.000 ω_2_ = 1.000	6 V(0.969) 81S(0.993) 179F(0.956)	1.000
	Null	−7771.13		*P*_0_ = 0.266 *P*_1_ = 0.028 *P*_2a_ = 0.639 *P*_2b_ = 0.067 ω_0_ = 0.040 ω_1_ = 1.000 ω_2_ = 1.000		
*nad1*	Alternative	−10505.00	2.06	*P*_0_ = 0.793 *P*_1_ = 0.046 *P*_2a_ = 0.151 *P*_2b_ = 0.009 ω_0_ = 0.023 ω_1_ = 1.000 ω_2_ = 2.826	50P(0.994) 113R(0.985) 162A(0.987) 223S(0.955) 227F(0.950) 239 M(0.965)	0.151
	Null	−10506.03		*P*_0_ = 0.798 *P*_1_ = 0.045 *P*_2a_ = 0.149 *P*_2b_ = 0.008 ω_0_ = 0.020 ω_1_ = 1.000 ω_2_ = 1.000		
*nad2*	Alternative	−9366.49	3.74	*P*_0_ = 0.247 *P*_1_ = 0.017 *P*_2a_ = 0.688 *P*_2b_ = 0.047 ω_0_ = 0.036 ω_1_ = 1.000 ω_2_ = 999.0	31E(1.000) 82 K(0.998) 149Y(1.000) 177G(0.994) 179P(1.000) 209Y(0.997)	0.053
	Null	−9368.36		*P*_0_ = 0.306 *P*_1_ = 0.021 *P*_2a_ = 0.630 *P*_2b_ = 0.043 ω_0_ = 0.034 ω_1_ = 1.000 ω_2_ = 1.000		
*nad3*	Alternative	−4357.38	0.06	*P*_0_ = 0.416 *P*_1_ = 0.037 *P*_2a_ = 0.502 *P*_2b_ = 0.045 ω_0_ = 0.016 ω_1_ = 1.000 ω_2_ = 1.936	21R(0.991)	0.806
	Null	−4357.41		*P*_0_ = 0.422 *P*_1_ = 0.037 *P*_2a_ = 0.497 *P*_2b_ = 0.044 ω_0_ = 0.016 ω_1_ = 1.000 ω_2_ = 1.000		
*nad4*	Alternative	−4094.18	0	*P*_0_ = 0.156 *P*_1_ = 0.004 *P*_2a_ = 0.821 *P*_2b_ = 0.019 ω_0_ = 0.015 ω_1_ = 1.000 ω_2_ = 1.000	16 N(0.993) 38F(0.989) 69A(0.990) 84 K(0.993) 93 W(0.965)	1.000
	Null	−4094.18		*P*_0_ = 0.156 *P*_1_ = 0.004 *P*_2a_ = 0.821 *P*_2b_ = 0.019 ω_0_ = 0.015 ω_1_ = 1.000 ω_2_ = 1.000		
*nad4l*	Alternative	−3627.82	0.28	*P*_0_ = 0.193 *P*_1_ = 0.023 *P*_2a_ = 0.701 *P*_2b_ = 0.083 ω_0_ = 0.010 ω_1_ = 1.000 ω_2_ = 4.556	20E(0.998) 63S(0.953)	0.597
	Null	−3627.96		*P*_0_ = 0.194 *P*_1_ = 0.023 *P*_2a_ = 0.701 *P*_2b_ = 0.083 ω_0_ = 0.009 ω_1_ = 1.000 ω_2_ = 1.000		
*nad5*	Alternative	−20296.01	10.76	*P*_0_ = 0.214 *P*_1_ = 0.008 *P*_2a_ = 0.751 *P*_2b_ = 0.027 ω_0_ = 0.038 ω_1_ = 1.000 ω_2_ = 3.614	106A(0.991) 190S(1.000)	0.001
	Null	−20290.63		*P*_0_ = 0.771 *P*_1_ = 0.138 *P*_2a_ = 0.077 *P*_2b_ = 0.014 ω_0_ = 0.042 ω_1_ = 1.000 ω_2_ = 1.000		

## 4 Conclusion

The present study is the first to sequence, annotate, and characterize the complete mitogenome of the estuarine clam *P. amurensis* (Corbulidae, Myida), thereby providing a foundational genetic resource for understanding mitochondrial evolution within Corbulidae and the broader order Myida. The *atp8* gene in the mitogenome of *P. amurensis* was successfully annotated, despite being frequently absent or difficult to detect in many bivalve mitogenomes due to its high sequence divergence and variable length. The results confirmed the presence of *atp8* in Corbulidae, and phylogenetic analyses based on 12 concatenated PCGs robustly recovered Corbulidae as the sister group to the Myidae–Dreissenidae clade. Molecular dating further indicated that *P. amurensis* diverged during the Middle Jurassic, which coincided with major episodes of marine ecological turnover. Furthermore, although purifying selection predominates across the mitogenome, branch‐site analyses revealed significant positive selection acting on *nad5*, with two lineage‐specific amino acid substitutions that likely enhance proton translocation efficiency under the osmotic variability characteristic of estuarine habitats. Such adaptations may confer resilience to osmotic stress and salinity fluctuations in estuarine environments, where ion regulation and energy metabolism play critical roles in survival. Overall, our findings substantially expand the available genomic resources for evolutionary investigations of bivalves and provide strong evidence that patterns of mitogenomic evolution are closely associated with ecological adaptation. Future studies integrating broader sampling of Corbulidae taxa and functional assays will be essential for validating the adaptive significance of the identified *nad5* mutations and elucidating the molecular mechanisms linking mitochondrial function to habitat specialization.

## Author Contributions

**Xuyi Yang:** conceptualization (lead), data curation (lead), formal analysis (lead), funding acquisition (lead), investigation (lead), methodology (lead), software (lead), visualization (lead), writing – original draft (lead). **Yalin Feng:** investigation (equal), methodology (equal), resources (equal), software (equal), visualization (equal). **Liang Xu:** data curation (equal), formal analysis (equal), resources (equal), visualization (equal). **Zan Liu:** investigation (equal), software (equal), validation (equal). **Tinghui Xie:** software (equal), validation (equal). **Zefeng Yu:** investigation (supporting), visualization (supporting). **Junyuan Li:** funding acquisition (lead), methodology (equal), project administration (lead), writing – review and editing (lead).

## Funding

This study was supported by the Taizhou Science and Technology Plan Project (No. 25nya01).

## Conflicts of Interest

The authors declare no conflicts of interest.

## Supporting information


**Figure S1:** Specimen photograph for *Potamocorbula amurensis* . The images were photographed by Xuyi Yang.
**Figure S2:** Phylogenetic tree inferred from the partitioned nucleotide sequences of 12 mitochondrial PCGs based on the Bayesian and maximum‐likelihood methods. The numbers near each node are Maximum likelihood bootstrap support values based on 1000 ultrafast bootstrap replicates in IQ‐tree and Bayesian inference posterior probabilities.
**Table S1:** Test of substitution saturation.
**Table S2:** Best partitioning scheme and substitution models selected by ModelFinder in this study.
**Table S3:** Fossil constraints used in the MCMCtree analyses in this study.
**Table S4:** RSCU analysis of protein coding region in *P. amurensis*. Note: * Relative synonymous codon usage, RSCU.
**Table S5:** Selective pressure analyses on the 12 concatenated mitochondrial protein‐encoding genes.

## Data Availability

The genome sequence data that support the findings of this study are openly available in GenBank of NCBI at [https://www.ncbi.nlm.nih.gov] under the accession no. PX644768. The associated BioProject, BioSample and SRA numbers are PRJNA1329176, SAMN51388399, and SRR35434659, respectively.
