# Supplementary material for: The Complete Mitogenome of the Estuarine Clam Potamocorbula amurensis (Corbulidae, Myida) and Its Implications for Phylogeny and Adaptation
Source: Ecol Evol. 2026 Jun 30;16(7):e73937. doi: 10.1002/ece3.73937 (PMC13318508; doi:10.1002/ece3.73937)
Supplement: Supplementary file 1 — Figure S1: Specimen photograph for Potamocorbula amurensis . The images were photographed by Xuyi Yang. Figure S2: Phylogenetic tree inferred from the partitioned nucleotide sequences of 12 mitochondrial PCGs based on the Bayesian and maximum‐likelihood methods. The numbers near each node are Maximum likelihood bootstrap support values based on 1000 ultrafast bootstrap replicates in IQ‐tree and Bayesian inference posterior probabilities. Table S1: Test of substitution saturation. Table S2: Best partitioning scheme and substitution models selected by ModelFinder in this study. Table S3: Fossil constraints used in the MCMCtree analyses in this study. Table S4: RSCU analysis of protein coding region in P. amurensis. Note: * Relative synonymous codon usage, RSCU. Table S5: Selective pressure analyses on the 12 concatenated mitochondrial protein‐encoding genes. [file ECE3-16-e73937-s001.docx]

**
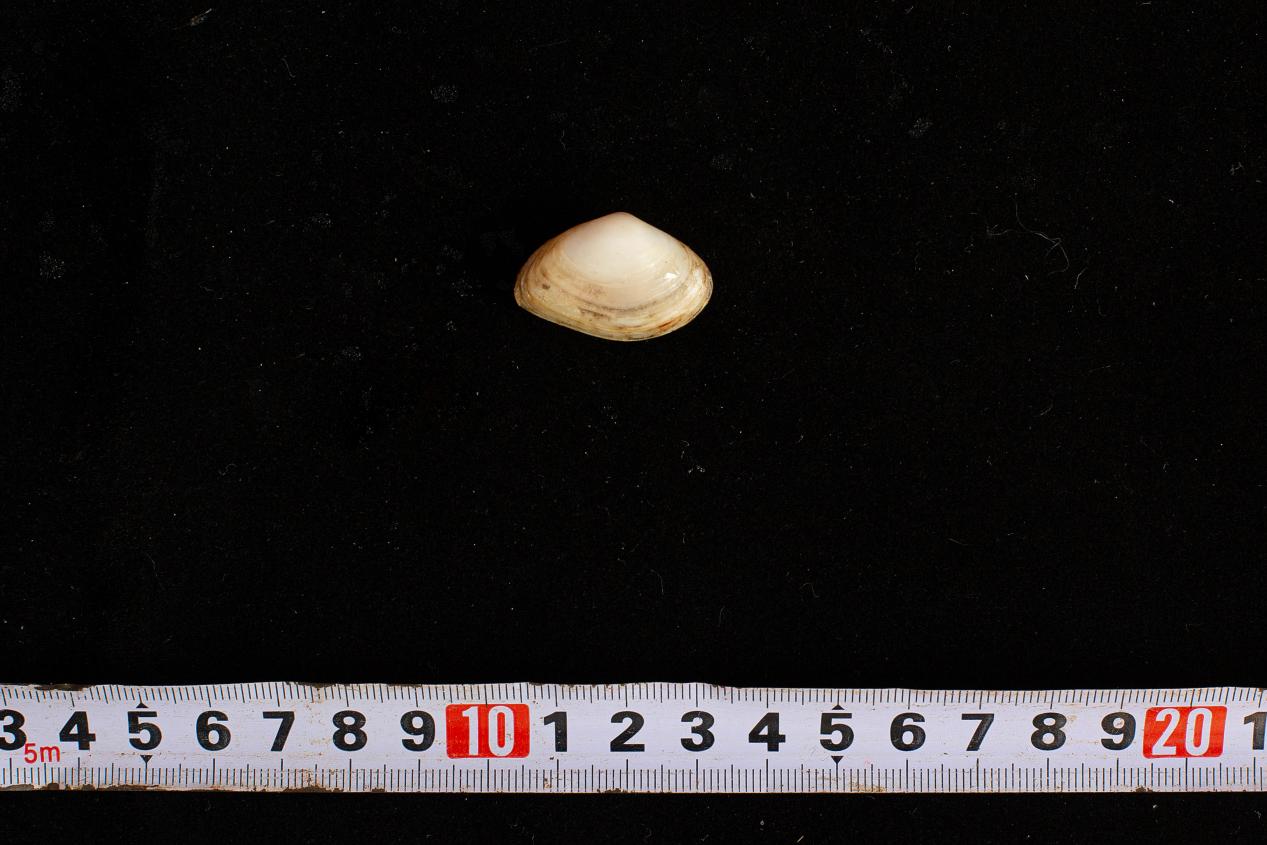
**

**Figure S1.** Specimen photograph for *Potamocorbula amurensis*. The images were photographed by Xuyi Yang.

**
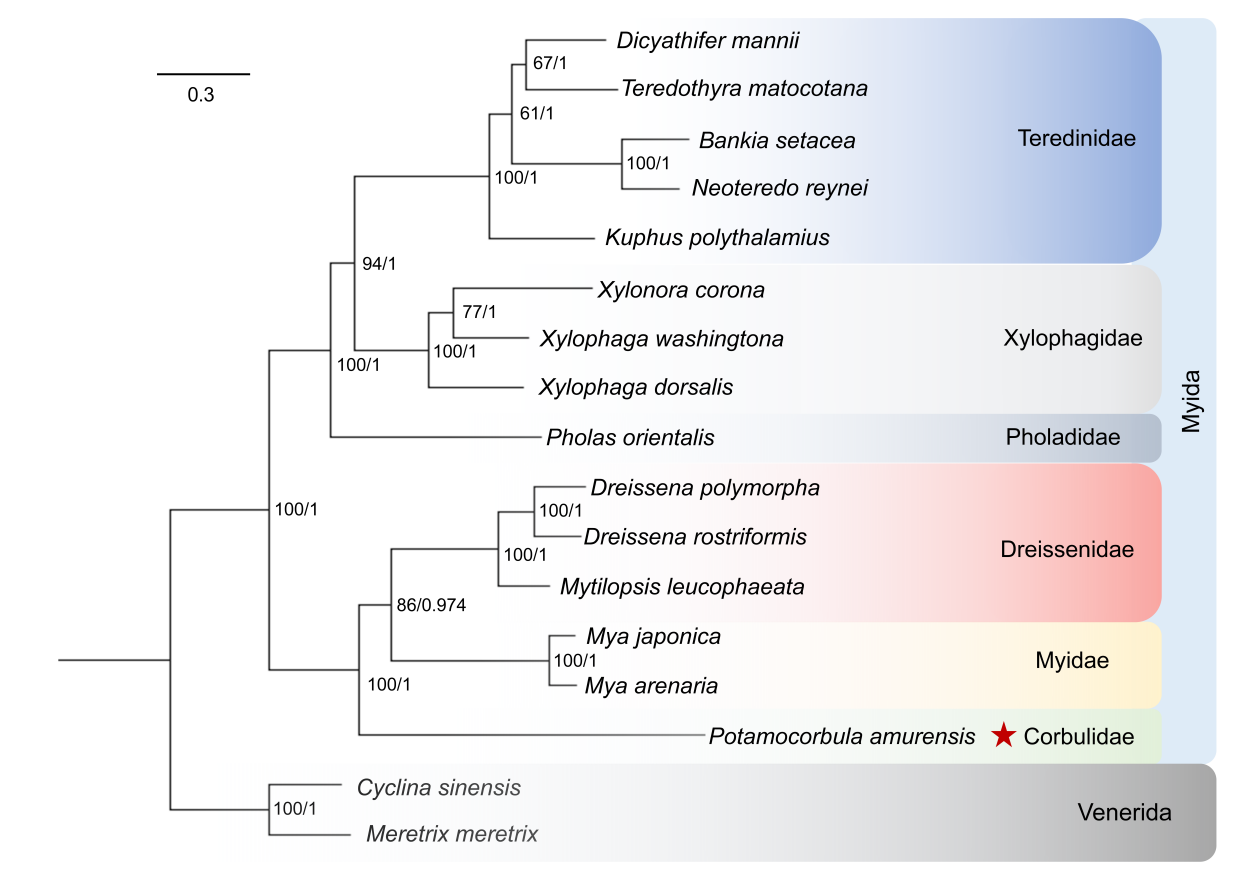
**

**Figure S2.** Phylogenetic tree inferred from the partitioned nucleotide sequences of 12 mitochondrial PCGs based on the Bayesian and maximum-likelihood methods. The numbers near each node are Maximum likelihood bootstrap support values based on 1000 ultrafast bootstrap replicates in IQ-tree and Bayesian inference posterior probabilities.

**Table S1.** Test of substitution saturation.

| Prop. invar. sites | 0.0000 |
| --- | --- |
| Mean H | 1.1005 |
| Standard Error | 0.0068 |
| Hmax | 1.7325 |
| Iss | 0.6352 |
| Iss.c | 0.8417 |
| T | 30.1819 |
| DF | 10256 |
| Prob (Two-tailed) | 0.0000 |
| 95% Lower Limit | 0.6218 |
| 95% Upper Limit | 0.6486 |

**Table S2.** Best partitioning scheme and substitution models selected by ModelFinder in this study.

| PCGs | Partition delineation | Best Model |
| --- | --- | --- |
| *atp6* | 1-660 | GY+F+R3 |
| *cox1* | 661-2166 | GY+F+I+I+R4 |
| *cox2* | 2167-2871 | GY+F+R5 |
| *cox3* | 2872-3675 | GY+F+I+I+R5 |
| *cytb* | 3676-4788 | GY+F+I+I+R7 |
| *nad1* | 4789-5679 | GY+F+R6 |
| *nad2* | 5680-6627 | GY+F+R10 |
| *nad3* | 6628-6984 | GY+F+R4 |
| *nad4* | 6985-8256 | GY+F+I+I+R3 |
| *nad4l* | 8257-8523 | GY+F+R5 |
| *nad5* | 8524-10125 | GY+F+I+I+R4 |
| *nad6* | 10126-10557 | GY+F+R3 |

**Table S3.** Fossil constraints used in the MCMCtree analyses in this study.

| Fossil constraints | upper limit (Ma) | lower limit (Ma) | References |
| --- | --- | --- | --- |
| Venerida | 339.4 | 336 | M’Coy, 1847; Wang et al, 2023 |
| Pholadidae and Teredinidae | 189.6 | 183 | Velazco, 2008 |

**References:**

M’Coy F. XXVIII.—On the fossil botany and zoology of the rocks associated with the coal of Australia. Annals and Magazine of Natural History, 1847, 20(134): 298–312.

Velazco YP. Some invertebrate fossils from Huacrapuquio, province of Huancayo, Junin, Peru. Revista Del Instituto De Investig De La Facultad De Minas, 2008, 11: 39–48.

Wang Y, Yang Y, Kong L, Sasaki T, Li Q. Phylogenomic resolution of Imparidentia (Mollusca: Bivalvia) diversification through mitochondrial genomes. Marine Life Science & Technology, 2023, 5(3): 326–336.

**Table S4.** RSCU analysis of protein coding region in *P. amurensis.* Note: * Relative synonymous codon usage, RSCU.

| Amino acid | Codon | Number | RSCU* | Amino acid | Codon | Number | RSCU* |
| --- | --- | --- | --- | --- | --- | --- | --- |
| Phe | UUU | 466 | 1.68 | Tyr | UAU | 173 | 1.62 |
|  | UUC | 88 | 0.32 |  | UAC | 40 | 0.38 |
| Leu2 | UUA | 121 | 2.24 | His | CAU | 15 | 1.30 |
|  | UUG | 133 | 2.46 |  | CAC | 8 | 0.7 |
| Leu1 | CUU | 47 | 0.87 | Gln | CAA | 13 | 1.18 |
|  | CUC | 9 | 0.17 |  | CAG | 9 | 0.82 |
|  | CUA | 9 | 0.17 | Asn | AAU | 114 | 1.68 |
|  | CUG | 5 | 0.09 |  | AAC | 22 | 0.32 |
| Ile | AUU | 109 | 1.64 | Lys | AAA | 73 | 1.18 |
|  | AUC | 24 | 0.36 |  | AAG | 51 | 0.82 |
| Met | AUA | 46 | 1.00 | Asp | GAU | 52 | 1.58 |
|  | AUG | 46 | 1.00 |  | GAC | 14 | 0.42 |
| Val | GUU | 157 | 2.20 | Glu | GAA | 30 | 0.90 |
|  | GUC | 41 | 0.57 |  | GAG | 37 | 1.10 |
|  | GUA | 34 | 0.48 | Cys | UGU | 134 | 1.49 |
|  | GUG | 54 | 0.76 |  | UGC | 46 | 0.51 |
| Ser2 | UCU | 119 | 1.80 | Trp | UGA | 86 | 0.95 |
|  | UCC | 52 | 0.79 |  | UGG | 95 | 1.05 |
|  | UCA | 60 | 0.91 | Arg | CGU | 12 | 1.50 |
|  | UCG | 37 | 0.56 |  | CGC | 3 | 0.38 |
| Pro | CCU | 25 | 2.56 |  | CGA | 7 | 0.88 |
|  | CCC | 7 | 0.72 |  | CGG | 10 | 1.25 |
|  | CCA | 6 | 0.62 | Ser1 | AGU | 97 | 1.47 |
|  | CCG | 1 | 0.10 |  | AGC | 31 | 0.47 |
| Thr | ACU | 40 | 1.70 |  | AGA | 62 | 0.92 |
|  | ACC | 13 | 0.55 |  | AGG | 96 | 1.43 |
|  | ACA | 27 | 1.15 | Gly | GGU | 79 | 1.65 |
|  | ACG | 14 | 0.60 |  | GGC | 29 | 0.60 |
| Ala | GCU | 54 | 2.20 |  | GGA | 29 | 0.60 |
|  | GCC | 19 | 0.78 |  | GGG | 55 | 1.15 |
|  | GCA | 16 | 0.65 |  |  |  |  |
|  | GCG | 9 | 0.37 |  |  |  |  |

**Table S5.** Selective pressure analyses on the 12 concatenated mitochondrial protein-encoding genes.

| Branch Model | np | lnL | Parameter estimates | Model compared | 2∆lnL |
| --- | --- | --- | --- | --- | --- |
| M_1_ (free-ratio model) | 65 | -124330.73 |  | M_1_ versus M_0_  M_1_ versus M_2_ | 1,007.96*  967.32* |
| M_2_ (two-ratio model) | 35 | -124814.39 | ω_0_ = 0.0258 ω_1_ = 0.1640 | M_2_ versus M_0_ | 40.64* |
| M_0_ (one-ratio model) | 34 | -124834.71 | ω = 0.0259 |  |  |
